# Type II Opsins in the Eye, the Pineal Complex and the Skin of *Xenopus laevis*: Using Changes in Skin Pigmentation as a Readout of Visual and Circadian Activity

**DOI:** 10.3389/fnana.2021.784478

**Published:** 2022-01-21

**Authors:** Gabriel E. Bertolesi, Nilakshi Debnath, Hannan R. Malik, Lawrence L. H. Man, Sarah McFarlane

**Affiliations:** Department of Cell Biology and Anatomy, Hotchkiss Brain Institute and Alberta Children’s Hospital Research Institute, University of Calgary, Calgary, AB, Canada

**Keywords:** melanophore, melanocyte, background adaptation, circadian rhythm, evolution, pigment cell, light response, photosensitive

## Abstract

The eye, the pineal complex and the skin are important photosensitive organs. The African clawed frog, *Xenopus laevis*, senses light from the environment and adjusts skin color accordingly. For example, light reflected from the surface induces camouflage through background adaptation while light from above produces circadian variation in skin pigmentation. During embryogenesis, background adaptation, and circadian skin variation are segregated responses regulated by the secretion of α-melanocyte-stimulating hormone (α-MSH) and melatonin through the photosensitivity of the eye and pineal complex, respectively. Changes in the color of skin pigmentation have been used as a readout of biochemical and physiological processes since the initial purification of pineal melatonin from pigs, and more recently have been employed to better understand the neuroendocrine circuit that regulates background adaptation. The identification of 37 type II opsin genes in the genome of the allotetraploid *X. laevis*, combined with analysis of their expression in the eye, pineal complex and skin, is contributing to the elucidation of the role of opsins in the different photosensitive organs, but also brings new questions and challenges. In this review, we analyze new findings regarding the anatomical localization and functions of type II opsins in sensing light. The contribution of *X. laevis* in revealing the neuroendocrine circuits that regulate background adaptation and circadian light variation through changes in skin pigmentation is discussed. Finally, the presence of opsins in *X. laevis* skin melanophores is presented and compared with the secretory melanocytes of birds and mammals.

## Introduction

Physiologically, color change of the skin is a critical process for survival in ectothermic amphibians. For example, during camouflage through background adaptation, the light reflected from the surface is sensed and the skin undergoes a color change to avoid detection by potential predators or prey (Hoekstra, 2006). The environmental light is also perceived in a circadian manner and the skin adjusts its color for heat retention and/or for light/ultraviolet (UV) protection (Filadelfi and Castrucci, 1996; Rudh and Qvarnström, 2013). Several physiological processes associated with skin color change are regulated by the neuroendocrine system. The skin itself also functions as a photosensitive organ, and distribution and synthesis of colored pigment also occur in cultures of skin cells (Oshima, 2001). For more than a century, the African clawed frog *Xenopus laevis* has been employed as a model organism to understand cellular and developmental biology (Dawid and Sargent, 1988). The changes in skin color are an important tool for revealing the neuroendocrine circuits that regulate background adaptation and circadian variations. The *Xenopus* model allowed elucidation of the “effectors” that change pigmentation, with the dispersing agent alpha-melanocyte stimulating hormone (α-MSH) darkening the skin during background adaptation, and melatonin lightening the skin at night (Filadelfi and Castrucci, 1996; Roubos et al., 2010; Bertolesi and McFarlane, 2018). Unknown, however, is the identity of the “initiators” that trigger the physiological color response; molecules that sense light in photosensitive organs such as the eye, the pineal complex (named here as the frontal organ plus the pineal gland) and the skin. Fortunately, two critical events in the last decade positioned *Xenopus laevis* as an excellent model organism to unveil the initiating components: (1) Full sequencing of the genome (Session et al., 2016), and (2) Discovery of a remarkable number and diversity of type II opsin photopigments which were originally detected in zebrafish (Davies et al., 2015) and compared with other species, including *Xenopus laevis* (Davies et al., 2015; Bertolesi et al., 2020).

Anurans (frogs), the amphibian order to which *Xenopus laevis* belongs, offer additional advantages in both broadening our understanding of the evolutionary processes that may have occurred during the transition to land, and providing information about the mechanisms of sensing light that produce changes in skin pigmentation. During evolution, the transition from aquatic to terrestrial life occurred together with dramatic changes in anatomical structures, sensory systems, and the skin. For example, developing limbs transformed the swimming locomotive organs to structures better adapted to land (Dickson et al., 2021), while the eye increased in size and moved from a lateral to a dorsal position characteristic of the early amphibian Tetrapod (Nilsson, 2021). Interestingly, the “buena vista” theory, recently suggested by MacIver et al. (2017), postulates that a contributing factor to the further evolution of limbs and the emergence of novel neurological circuits was the evolution of a sensory system capable of “seeing” over longer distances, which preceded the evolution of fully terrestrial limbs. The formation of limbs during metamorphosis from swimming tadpoles to land-adapted froglets, the change in the anatomical position of the eye from a lateral to dorsal position, and the generation of novel neuronal circuits are important features of Anuran development that show certain similarities with the evolutionary processes. These evolutionary-developmental similarities are evident in Anurans of the other two orders of living amphibians: the Caudata (salamanders) and Gymnophiona (caecilians). The evolution and photoreception of the three orders of amphibians were reviewed recently (Mohun and Davies, 2019). In this review we focus on *Xenopus laevis* and emphasize skin pigmentation changes that occur during early development (see section “Changes in Skin Pigmentation During Early Development. Background Adaptation and Circadian Variation in Skin Response Are Segregated and Driven by Photosensitivity of the Eye and the Pineal Complex, Respectively”). The evolutionary origin of this allotetraploid species, and the variability of type II opsins and their anatomical expression in the eye, the pineal complex, and the skin are discussed in section “Origin of *Xenopus laevis* and Genetics of Type II Opsins.”

Using changes in skin pigmentation as a readout of light-sensing mechanisms is simple in *Xenopus*, at least during early development, in that the eye and the pineal complex are both photosensitive, and the neuroendocrine circuits that induce skin color variation are segregated (Bertolesi et al., 2020). Mammals are more complicated when it comes to understanding the light-regulated pigmentation processes, since their ancestors lost an external photosensitive pineal complex as it became incorporated into the brain. In mammals, neuroendocrine light-mediated changes in skin pigmentation are under the control of eye photosensitivity alone (Klein, 2006), which prevents the separate analysis of skin color changes triggered by visual (e.g., background adaptation) and non-visual (e.g., circadian) processes. The extensive nocturnal period which mammalian ancestors embraced in order to avoid competing with the diurnal and dominant reptiles [nocturnal bottleneck theory (Gerkema et al., 2013)] may have been the selective force for the loss of an external photosensitive pineal complex. Additionally, the evolution of insulation systems that appeared independently during the advent of thermoregulation in avian-reptile and mammalian lineages induced dramatic changes in the characteristics of the integument, by transforming intracellular pigment-dispersing melanophores into pigment-secretory melanocytes (Haslam et al., 2014; Lovegrove, 2017). Thus, *Xenopus* possess a pigmentation system that responds to signals from several photosensitive organs. In section “Function of Type II Opsins in *Xenopus* eye. The Role of Intrinsically Photosensitive Horizontal Cells (ipHCs) and Pinopsin-Expressing Photoreceptors” we analyze the structure of the *Xenopus* eye, its activation during development, and the expression of type II opsins in the context of their visual role to regulate background adaptation. The anatomy of the pineal complex as a photosensor of environmental light is discussed in section “Structure of the Pineal Complex, Expression of Type II Opsins and Melatonin Secretion.” Finally, in section “Type II Opsins in the Skin. From the Ectothermic *Xenopus* to Endothermic Mammals,” the presence of opsins in *X. laevis* skin melanophores is compared to our current knowledge of the involvement of opsins in secretory melanocytes of mammals.

### Changes in Skin Pigmentation During Early Development. Background Adaptation and Circadian Variation in Skin Response Are Segregated and Driven by Photosensitivity of the Eye and the Pineal Complex, Respectively

**FIGURE 1:**
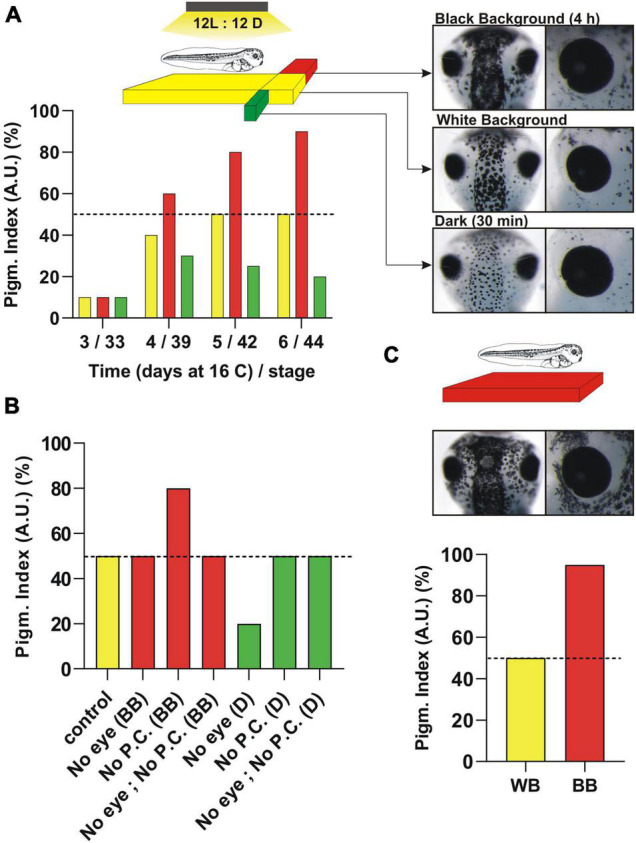
Pigmentation response induced by reflected light from the substrate (background adaptation) or environmental light (dark/light) over *Xenopus laevis* development. **(A)** Pigmentation levels at different developmental times represented approximately (50%; day 5/6) from embryos raised on a white background (WB) (yellow column). The light shines from above in cycles of 12 h ON/12 h OFF and the pigmentation is measured at the middle of the light cycle. The timeline in days (*X*-axis) of embryos maintained at 16°C until stage 43/44 is assigned to the approximate developmental stage. Tadpoles raised on a white substrate (WB) are switched either to a black background for 4 h (BB; red columns) or dark for 30 min (D; green columns) (see schematic). The pigmentation response is measured in the dorsal head (see pictures). **(B)** Pigmentation response of stage 43/44 embryos compared to enucleation (No eye), pinealectomy (No P.C) or both (No eye, No P.C.). The surgery is performed 24 h before pigmentation measurement. **(C)** Morphological pigmentation of embryos induced by raising tadpoles on a black background from early stages. Note the difference in the “morphological” pigmentation (increase in melanophore number mainly around the eye) when tadpoles are raised on a BB vs. the quick “physiological” responses seen in tadpoles raised on a WB. See text for additional details.

The integument of amphibians, such as the frog *Xenopus laevis*, non-avian reptiles, and fish, is exposed to light and rapidly adapts its pigmentation color to the local environment. Conversely, avian reptiles and mammals possess an integument protected by feathers and fur, respectively, and display markedly slower color changes. In *Xenopus* larvae, color change is fast, with two physiological responses observed in minutes or hours if the larvae are raised initially on a white background with light shining from above: (1) Background adaptation, and (2) Circadian variation in skin color. Switching the larvae from a white to a black background darkens the skin by the dispersion of melanosomes (melanin-filled vesicles) residing in dermal pigment cells called melanophores (Figure 1A). This cryptic physiological response, termed background adaptation, increases survival by reducing detection by natural predators. The response was described in *Xenopus* in some detail almost a century ago by Hogben and Slome (1931, 1936). Indeed, the authors were the first to indicate that background adaptation is mediated by a neuroendocrine circuit that requires a functional hypophysis, specifically the pars intermedia. Work, years later, demonstrated that synthesis and secretion of the pigment dispersing agent α-MSH from the hypophysis is responsible for background adaptation in amphibians (Jenks et al., 1977; Kramer et al., 2003).

In contrast to black background adaptation, when tadpoles are switched to dark conditions melanosomes aggregate and lighten the skin (Figure 1A). Although the skin lightening response in dark conditions was known for several years (McCord and Allen, 1917), it was not until the mid 20th century when a “chemical substance” released from the pineal gland was recognized as an “effector” that induces melanosome aggregation (Bagnara, 1960a). Melatonin was biochemically purified from pig pineal glands, helped by the lightening of tadpole skin as a readout (Lerner et al., 1958). It is worth noting that all vertebrates, independent of whether they are diurnal or nocturnal, release melatonin into the bloodstream at night. In *Xenopus*, the circadian whitening of skin by melatonin is thought to contribute to thermoregulation (Filadelfi and Castrucci, 1996).

To understand the neuroendocrine circuits responsible for background adaptation and the circadian variation in skin color, our laboratory and others focused on the early developmental window and determined when the responses initiate (Jenks et al., 1977; Verburg-van Kemenade et al., 1984; Bertolesi et al., 2017). An advantage of *Xenopus laevis* is the simplicity of the pigmentation system in that of the eight different types of colored pigmented chromatophore described in vertebrates (brown melanophores, red erythrophoros, blue cyanophores, yellow xanthophores, white leucophores, reflective iridophore and the dichromatic erythro-iridophore and erythro-cyanophore) (Schartl et al., 2016), only brown, melanin-containing melanophores are present at early developmental times (Fukuzawa, 2000; Kumasaka et al., 2005). Pigment cells are derived from the neural crest, which proliferate and differentiate–with pigment cells being visible by the naked eye over the dorsal head, belly and tail by stage 32–33/34 (day 3 at 16°C) in *Xenopus* tadpoles. Slight differences in pigmentation are observed that depend on the level between different hatches. The response to black background emerges at stage 39/40 (Figure 1A; day 4 at 16°C), immediately after the eye circuits becomes functional (stage 37; see section “Function of Type II Opsins in *Xenopus* eye. The Role of Intrinsically Photosensitive Horizontal Cells (ipHCs) and Pinopsin-Expressing Photoreceptors”) (Verburg-van Kemenade et al., 1984; Bertolesi et al., 2014, 2017). The change in pigmentation induced by black background becomes more apparent within 2 days (Figure 1A, day 5 and 6 at 16°C; stage 42 and 43/44). Skin lightening triggered by dark also initiates at stage 39, but only if embryos are raised on a light/dark cycle (Figure 1A). Raising tadpoles in continuous light or dark delays the onset of the circadian light response (Bertolesi et al., 2017), likely because of lack of entrainment of the pineal complex.

Two important questions that needed addressing were whether: (1) background adaptation and circadian skin color variation are developmentally segregated, and (2) skin pigmentation can be used as a readout of both of the photosensitive circuits. The schematic in Figure 1 shows the relative skin pigmentation of embryos developed on a white background with light shining down from above in light cycles of 12 h ON: 12 h OFF which produces an “intermediate” level of skin pigmentation when measured at the middle of the light cycle (Figures 1A–C, exemplified as 50% pigmentation level). This intermediate pigmentation level is an advantage as it allows quantifiable darkening and lightening responses to changes in background and environmental light, respectively. Thus, when tadpoles are switched to either a black background for approximately 3–4 h, or dark conditions for 30 min, the skin darkens or lightens, respectively (Figure 1A, schematic). These numbers correspond to the amount of time required to induce the maximum response (Bertolesi et al., 2017). When embryos reach stage 43/44, both neuroendocrine circuits are functional and both darkening and lightening are easily measurable – mainly in the dorsal head (Figure 1A, pictures). Pigmentation changes are also detectable in the belly and the tail (Bertolesi et al., 2015). Of note, the process of aggregation/dispersion of pigment over short time periods described here is known as “physiological color change” and differs from the “morphological” variation in pigmentation. The latter response comprises a slow change in color that occurs generally by varying the number of pigment cells and/or the type of pigment (Sugimoto, 2002; Bertolesi et al., 2016a). Morphological variation in skin pigmentation is detectable in *Xenopus* larvae raised on a black background from early stages when compared to larvae raised on a white background, with the black background increasing the number of melanophores (Bertolesi et al., 2016a; Figure 1C, morphological pigmentation; see the increased number of melanophores around the eye).

We recently showed that both neuroendocrine circuits are functional by stage 43/44 (Bertolesi et al., 2020). At this stage, the circuits are independent of each other, with background light being sensed by the eye and environmental light detected by the photosensitive pineal complex. Indeed, with eye enucleation the melanosome dispersion triggered by 4 h on a black background disappears, while the lightening induced by dark exposure remains similar to that of sham controls (Figure 1B). In contrast, pinealectomy abolishes the lightening triggered by dark conditions, but not the background adaptation response (Figure 1B; Bertolesi et al., 2020). Of note, pinealectomy in young embryos removes the secretory structure responsible for systemic melatonin (see section “Structure of the Pineal Complex, Expression of Type II Opsins and Melatonin Secretion”), so a simple experiment, where the pineal complex was covered with a small piece of aluminum foil, was used to show that the organ is photosensitive and responsible for the skin lightening induced by dark (Bertolesi et al., 2020).

An additional aspect to consider when skin pigmentation is used as a readout is the capacity of melanophores themselves to sense light and trigger either the dispersion or aggregation of melanosomes. This process, discussed in section “Type II Opsins in the Skin. From the Ectothermic *Xenopus* to Endothermic Mammals,” is known as the “primary color response” and occurs when photosensitive molecules, like type II opsins, initiate a signal transduction cascade that affects the distribution of melanosomes (Oshima, 2001). *Xenopus* melanophores *in vitro* exhibit primary responses (Seldenrijk et al., 1979; Daniolos et al., 1990; Rollag, 1996). The fact that pigmentation in the enucleated and pinealectomized larvae is similar to that of sham controls (Figure 1B; Bertolesi et al., 2020), however, indicates that the primary response *in vivo* has a minimal role when compared to the pigmentation changes induced by the “secondary” responses triggered by hormones; α-MSH for background adaptation and melatonin for environmental light.

Since background adaptation and circadian responses are segregated at the circuit level, and the primary response of melanophores provides a negligible contribution, changes in skin pigmentation can be used to distinguish signals that originate independently in the eye and the pineal complex. Interesting questions remain: Are these results applicable to older developmental stages of *Xenopus*, and further, to other species? Unfortunately, experiments that compare simultaneously the pigmentation response with enucleation or pinealectomy have not been performed in older post-metamorphic froglets. Neural circuits are known to change over development, and so it is not a given that the pigmentation response would be controlled similarly in embryos and adults. For instance, new neuronal connections are made by the eye just prior to metamorphosis, as per the generation of the axons of ipsilateral retinal ganglion cells to complement the contralateral axon projection formed at earlier developmental times (Larsson, 2011; von Uckermann et al., 2016). Ipsilateral and contralateral connections from both eyes provide stereoscopic vision. Little is known with respect to neuronal interactions between the photosensitive pineal complex and the eye in adult *Xenopus*, although new connections are not unexpected.

In considering whether other species have similar pigmentation responses to *Xenopus* it is important to bear in mind the dramatic changes that occurred during evolution in the mechanisms that regulate skin pigmentation. For example, background adaptation in Tetrapoda is regulated by a “uni-hormonal” mechanism, where changes in the systemic levels of the dispersing hormone α-MSH alter pigmentation color. In contrast, teleosts exhibit “dual hormonal” control, mediated by α-MSH and a second pituitary hormone with melanosome-aggregating ability known as melanin-concentrating hormone like (MCHL) (previously known as MCH) (Kawauchi et al., 1983; Bertolesi et al., 2019; Diniz and Bittencourt, 2019; Bertolesi and McFarlane, 2020). Additionally, the evolution of systems of insulation during the advent of thermoregulation modified the integument of avian-reptiles and mammals in such a way that places them in a different integument class relative to amphibians and non-avian reptiles (Lovegrove, 2017). Thus, additional species with mechanisms and observable color changes in the skin similar to those found in *Xenopus laevis* should likely be present within the amphibian and/or the reptile lineages.

### Origin of *Xenopus laevis* and Genetics of Type II Opsins

*Xenopus laevis* is an allotetraploid species that arose *via* the interspecific hybridization of diploid progenitors with 2n = 18, with the subsequent stabilization of the duplicated genome to restore meiotic paring. Thus, its total chromosome number (2n = 36) nearly doubles that of the Western clawed frog *Xenopus tropicalis* (2n = 20) [used as a reference genome (Session et al., 2016)] and the existent species in the *Xenopus* taxonomic genus known today (29 species). The full genome sequence is accessible through Xenbase^1^, and the chromosomal localization of genes is found in any of the pairs co-orthologous to the corresponding *X. tropicalis* chromosome, denoting an L and S for the longer and shorter homolog, respectively. The sex chromosome (Z/W) corresponds to 2L which includes the sex determinant gene, *dmw* (W-linked DM-domain), presented as a single allele but absent in males (Yoshimoto and Ito, 2011). In evolutionary terms, *X. laevis* is a relatively recent species in that the diploid progenitor diverged around 34 million years ago (mya) and combined to form the allotetraploid ancestor around 17–18 mya (Session et al., 2016).

**FIGURE 2:**
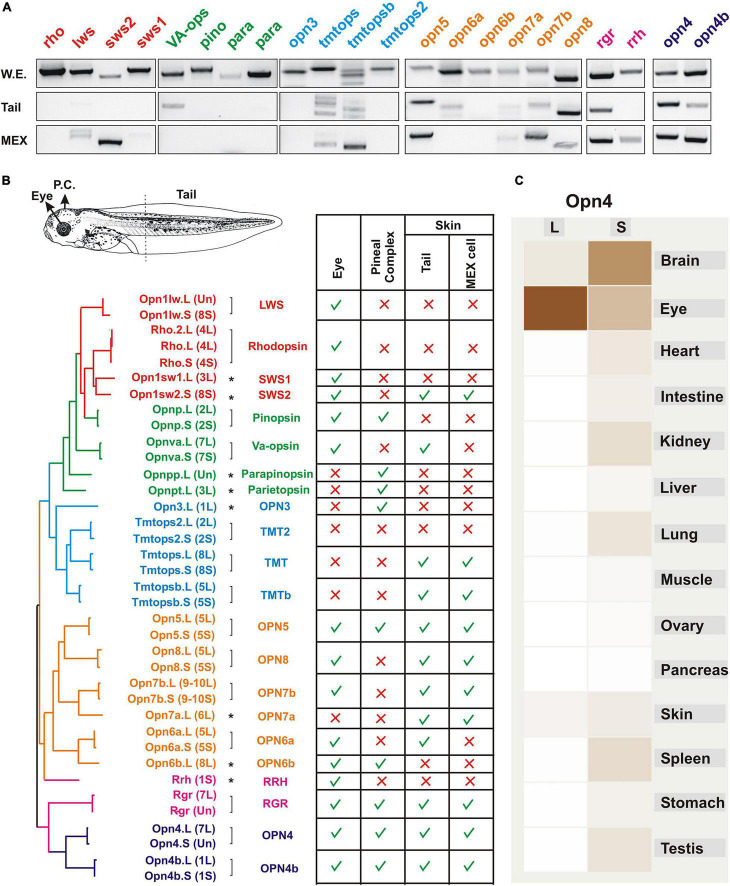
Expression of opsins in the *Xenopus* larval eye, pineal complex, and skin. **(A)** RT-PCR analysis showing mRNA expression of the indicated opsins at stage 43/44 in the whole embryo and isolated tails, and in a melanophore cell line (MEX) obtained from stage 30–35 *X. laevis* tadpoles (Kashina et al., 2004). RT-PCR conditions are similar to those published previously (Bertolesi et al., 2020, 2021). **(B)** Opsins identified in the *X. laev*is genome and their expression in the pineal complex, eye and tails. The putative amino acid sequences were compared, and a molecular phylogenetic analysis performed by the maximum-likelihood method is shown (not to scale). Type II opsins are grouped according to what was initially considered as their tissue localization: visual opsins (red); pineal opsins (green); non-visual opsins (blue); neuropsins (orange); photoisomerases (pink) and melanopsins (purple). Groups are also approximately reflected in the phylogenetic tree. Brackets denote genes duplicated and maintained on the long (L) and short (S) chromosomes, with single copy genes indicated with an asterisk. The table show the summary of expression of opsins in the eye (Bertolesi et al., 2021), the pineal complex (Bertolesi et al., 2020), and the tails and MEX cells as determined by RT-PCR as in **A.** **(C)** A comparative expression analysis determined by RNAseq of *opn4* in different organs from both genes (L and S) is reproduced from Xenbase (http://www.xenbase.org/, RRID:SCR_003280). Darker color reflects higher mRNA expression.

Our laboratory has focused on the identification of type II opsins in an effort to determine the light sensors that trigger changes in skin pigmentation. Of note, type II opsins possess a different evolutionary origin than the type I (microbial) opsins, although some similarities at the molecular level exist. Common to both opsin types are the seven transmembrane domains and the capacity to sense light to trigger an intracellular signal. However, type II opsins belong to a family of G-protein-coupled receptors (GPCRs) that likely derived from the melatonin receptor approximately 711–700 mya, before the appearance of vertebrates (approximately 540 mya) (Feuda et al., 2012; Porter et al., 2012). An amino acid switch to a lysine in the seventh transmembrane domain allowed the binding of a chromophore (“photosensor”; generally retinal) to trigger a conformational change and a G-protein-associated signal transduction cascade upon light stimulation (Lamb, 2013). We identified a total of 37 opsin genes in the *X. laevis* genome, most of which correspond to duplicated forms present in the L and S chromosomes, while others are single copies generated as a result of pseudogenization (Bertolesi et al., 2020; Figures 2A,B). Without considering duplicated genes, a total of 22 opsins are present, and we recently reported their expression in the stage 43/44 tadpole eye (Bertolesi et al., 2021) and pineal complex (Bertolesi et al., 2020; Figure 2B). As an approximation of opsins expressed by skin melanophores, we show here the RT-PCR analysis of opsins detected in isolated tails from stage 43/44 tadpoles, using similar PCR conditions to those published previously (Bertolesi et al., 2020, 2021; Figure 2A). We also analyzed the expression of opsins in a stable melanophore cell line generated from stage 30 to 35 tadpoles, MEX cells (Kashina et al., 2004; Figure 2A). The 22 opsins are classified in six phylogenetically related groups, named based on their tissue localization as identified in initial expression analyses, although current knowledge indicates a more complex scenario (Figure 2B): (1) the “**visual opsins**” [rhodopsin (*opn2*; Rh1); low wavelength sensitive opsin (*opn1lw*; Lws); short wavelength sensitive opsin 1 (*opn1sw1*; Sws1) and short wavelength sensitive opsin 2 (*opnsw2*; Sws2); (2) the “**pineal opsins,**” also known as “non-visual opsins” as they were identified in extraocular tissues of non-mammalian vertebrates [pinopsin (*opnp*); vertebrate ancient opsin (*opnva*), parapinopsin (*opnpp*) and pareietopsin (*opnpt*)]; (3) the “**non-visual opsins**” [encephalopsin (*opn3*) and several teleost multiple tissues opsins (*tmtops*; *tmtopsb* and *tmtops2*)]; (4) the “**neuropsins**” [opsin5 (*opn5;* is the only neuropsin gene present in mammals), and several paralogs identified in non-mammalian vertebrates (*opn6, 7*, and *8*); in this group, additional duplications occurred in *opn6* and *7* in *Xenopus* (*opn6a* and *opn6b*, and *opn7a* and *opn7b*)]; 5) the “**photoisomerases**” [the retinal G-protein coupled receptor (*rgr*) and retinal pigment epithelium (RPE)-derived rhodopsin homolog (*rrh*; a.k.a. peropsin)]; and (6) the “**melanopsins**” [*Xenopus* contains two paralogous genes, the mammalian-like *opn4* (a.k.a. *opn4m*) and the *Xenopus*-like *opn4b* (a.k.a. *opn4x*)]. Of the type II opsins described in vertebrates, those absent from the *Xenopus* genome are *exorhodopsin* and *opn9*, which appeared during evolution only in the teleost lineage, and the rhodopsin-like 2 gene (*rh2*) that was lost in amphibians and mammals (Davies et al., 2015).

Both the identification of type II opsins and analysis of their expression is proving helpful in determining their functional roles in regulating skin pigmentation. The genome duplication, however, remains an important consideration in that the two “subgenomes” have evolved asymmetrically, with one showing more intrachromosomal rearrangement, gene loss by deletion and pseudogenization than the other (Session et al., 2016). Indeed, expression of type II opsin genes varies between photosensitive organs and between the subgenomes as suggested by RNAseq analysis (Figure 2C). Of note, data regarding gene expression (RNAseq) and chromatin data (Chip-seq) are now integrated and available in Xenbase (Fortriede et al., 2020).

The anatomical localization of the Type II opsins in the eye, pineal complex and skin are discussed in the next three sections and provide important information for understanding the molecular mechanisms that regulate changes in skin pigmentation.

### Function of Type II Opsins in *Xenopus* Eye, the Role of Intrinsically Photosensitive Horizontal Cells and Pinopsin-Expressing Photoreceptors

**FIGURE 3:**
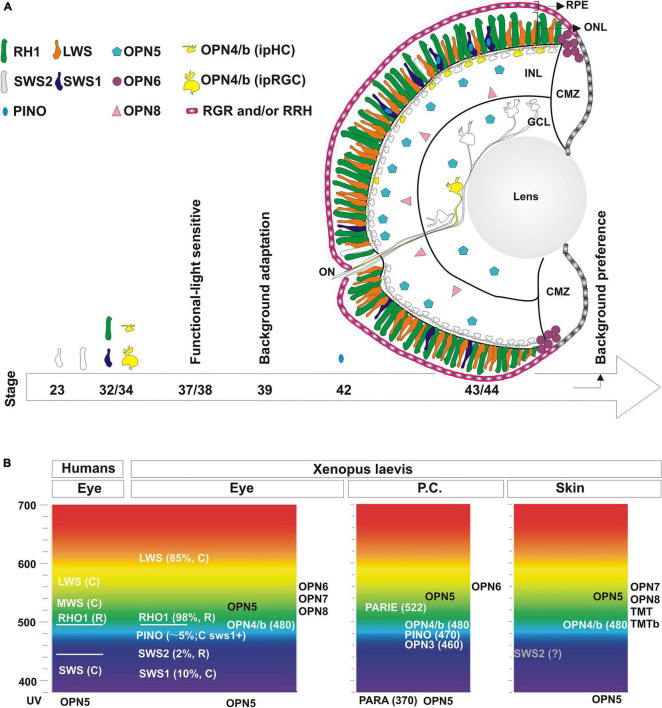
Localization and developmental expression of opsins in the eye. **(A)** Schematic of the central retina showing the expression of opsins in photoreceptors in the outer nuclear layer (ONL) in rods (Rh1; green and Sws2; white) and cones (Lws; orange and Sws1, blue). Pinopsin (light blue) is always co-expressed dorsally with a few *sws1* positive cells. Melanopsin (yellow) is expressed both by intrinsically photosensitive horizontal cells (ipHC) located dorsally in the retina in the inner nuclear layer (INL), and a minor population of retinal ganglion cells in the ganglion cell layer (GCL) (RGCs; 2% of total), called the intrinsically photosensitive RGCs (ipRGC). *opn5* mRNA (magenta) is expressed by cells in the outer portion of the INL and in the GCL, while *opn8* mRNA (pink) is present in cells in the inner part of the INL, likely amacrine cells. *opn6* (purple) expressing cells sit at the boundary of the proliferative CMZ and the ONL. *rgr* and *rrh* mRNAs (pink) are expressed by the RPE but excluded from the CMZ. A developmental timeline is schematized with an arrow at the bottom, with the emergence of opsin expression or specific behavioral responses indicated. **(B)** Opsins expressed in the eye, the pineal complex, and the skin (tails or MEX cells) of *Xenopus laevis* are shown at the approximate absorbance peak of their spectral sensitivity, and compared to the spectral sensitivity of human visual opsins. See text for additional details.

The *X. laevis* eye is well studied and the structure of the retina is similar to that found in all vertebrates. Within the retina, there are five classes of neurons distributed in three different layers: photoreceptors, horizontal, bipolar, amacrine, and retinal ganglion cells (Lamb, 2013). Anurans exhibit two types of classical photoreceptors, rods and cones, with specific characteristics regarding the expression of type II opsins (see below). Light entering the eye stimulates photoreceptors located in the outer nuclear layer (ONL), which transmit signals to bipolar cells and then retinal ganglion cells. The axons of retinal ganglion cells exit out of the eye and target the brain. Horizontal and amacrine cells sit in the inner nuclear layer (INL) and function to regulate signals within the retina (Figure 3A).

Rods and cones express type II opsins, and are almost equally represented and evenly distributed between the central and the peripheral retina of adults and young *Xenopus* larvae (Gábriel and Wilhelm, 2001; Bertolesi et al., 2021; Figure 3A). Intriguingly, in the majority of vertebrates there is only one kind of rod – the red rod - with the rest of the photoreceptors being cones. Anurans are unique in this respect for having both “red” and “green” rods. Rods constitute half of the total photoreceptors, and approximately 97–98% are the classical “red rods” which express the photopigment rhodopsin (Rh1) (Gábriel and Wilhelm, 2001), with the “green rods” expressing the short wavelength sensitive 2 (Sws2) opsin (Darden et al., 2003; Figure 3A). As for the cones, 85% contain the long-wavelength-sensitive opsin (Lws) and the remaining the short-wavelength-sensitive opsin 1 (Sws1) (Gábriel and Wilhelm, 2001; Bertolesi et al., 2021; Figure 3A). The Sws2 photopigment found in *Xenopus* green rods contributes to color vision (Kojima et al., 2017; Patel et al., 2020). While it is traditionally accepted that rods mediate night vision due to their high sensitivity to light, the Sws2 photopigment may contribute to both night sensitivity and color vision; Indeed, the isomerization characteristics of Sws2 allows the Sws2 rods to work together with red rods for low-light vision, while also providing color discrimination between gray and blue (Kojima et al., 2017). A third class of cones, “miniature” cones, were also detected in adult frogs that constitute less than 4% of the total percentage of all cones. UV sensitivity was intimated for these miniature cones based on a similar morphology of the *Xenopus* cones with those in the retina of salamanders (Zhang et al., 1994). However, we did not detect miniature cones or UV-sensitive opsins in photoreceptors of young *Xenopus* larvae (Bertolesi et al., 2021). Instead, we find a few cones (less than 5%) that express pinopsin, a pigment sensitive to blue wavelength light. Pinopsin was initially discovered in the pineal gland (Okano et al., 1994) and is postulated to regulate melatonin production (Natesan et al., 2002) (see section “Structure of the Pineal Complex, Expression of Type II Opsins and Melatonin Secretion”). Interestingly, in *X. laevis* larvae, pinopsin is co-expressed in approximately 50% of the *sws1* positive cells, mainly in the dorsal retina (Bertolesi et al., 2021; Figure 3A). In adult *X. tropicalis, sws1* positive cells as well as a small fraction of *rh1* positive cells co-express pinopsin (Sato et al., 2018a). Of note, pinopsin is found in other vertebrates, including non-avian reptiles (Taniguchi et al., 2001) and avian-reptiles, but only in the pineal complex for the latter (Okano et al., 1994). Pinopsin was lost during evolution in mammals and teleosts (Davies et al., 2015). Together, these data show that *Xenopus laevis* tadpoles exhibit several different photoreceptors that express distinctive type II opsins, including *rh1* or *sws*2 (rods), *lws*1 or *sws*1 (cones) and *sws1-pino* (co-expressing cones) (Figure 3A).

Melanopsin, another blue-sensitive opsin encoded by two paralogous genes (*opn4* and *opn4b*), was originally found in *Xenopus*, with expression reported in the eye, pineal complex, and melanophores (Provencio et al., 1998; Koyanagi et al., 2005). In the *Xenopus* retina, *opn4* and *opn4b* are co-expressed in a subset of horizontal and retinal ganglion cells (Bertolesi et al., 2015, 2021; Bertolesi and McFarlane, 2018; Figure 3A). Melanopsin confers these cells with photosensitivity, a fact that led to their being named intrinsically photosensitive horizontal (ipHCs) and retinal ganglion (ipRGCs) cells. In *Xenopus*, the ipHCs are located in the dorsal retina and are 6-7 times more abundant than the ipRGCs in the central retina, which constitute only 2% of the total ganglion cells (Bertolesi et al., 2014, 2015; Figure 3A). The L and S copies of the two melanopsin genes remain in the *Xenopus* genome. Of note, *opn4* and *opn4b* paralogs arose originally from a gene duplication 600 mya, but *opn4b* was lost in mammals due to unknown pressures of selection (Bellingham et al., 2006). Thus, during mammalian evolution both the *opn4b* gene as well as the ipHCs were lost, while ipRGCs remained.

The role of the ipHCs is not fully understood, but horizontal cells (HCs) as a general class have been studied extensively in many animal models including *Xenopus*. Studies pointed to the existence of ipHCs prior to their actual discovery. For instance, in Anurans HCs are classified based on their response to light: “Luminosity” HCs and “Chromaticity” HCs (Gábriel and Wilhelm, 2001). Luminosity HCs, like mammalian HCs, hyperpolarize to stimuli of all wavelengths, and provide inhibitory feedback to photoreceptors (Hassin and Witkovsky, 1983; Gábriel and Wilhelm, 2001). The feedback mechanism aids in fine-tuning contrast and color vision. Therefore, luminosity HCs likely correspond to melanopsin-negative HCs. Conversely, Anuran “chromaticity” HCs differentially respond to a variety of wavelengths and exhibit depolarizing responses (Stone et al., 1990). Interestingly, electrophysiological recordings from isolated ipHCs from teleosts and birds also indicate a light-induced depolarization response (Cheng et al., 2009; Sun et al., 2014; Morera et al., 2016), suggesting that the “chromaticity” HCs correspond to ipHCs (Figure 3A). Interestingly, we find differences between HCs and ipHCs with respect to the expression of the immediate gene marker, *c-fos*, in that the classical HCs turn on *c-fos* expression in response to light, while the ipHCs do not (Bertolesi et al., 2014). Melanopsin expression in ipHCs should make them light sensitive and give them a photosensing role. Evolutionarily, this is the most plausible scenario considering *opn4b* positive ipHCs in chicken act as photoreceptors (Morera et al., 2016). Further study is required, however, to understand the differences and the functional role of HCs and ipHCs in *Xenopus.*

In mammals, the only melanopsin gene, *opn4*, is expressed in 2% of retinal ganglion cells found primarily located in the dorsal retina, where it plays key roles in both visual and non-visual responses. Entrainment of the circadian rhythm and sleep latency are examples of non-visual functions (Hannibal et al., 2002; Hattar et al., 2002; Lupi et al., 2008). Melanopsin also has a visual role in the recognition of background light intensity and image representation (Storchi et al., 2017; Allen et al., 2019). Thus, it is possible that with the evolutionary loss in mammals of both the ipHCs and a photosensitive pineal gland, the ipRGCs, together with additional retinal circuits, took control of important visual and non-visual physiological functions.

Several neuropsins are expressed in the eye of *Xenopus*, although their physiological roles are not completely understood. Opn5, a UV sensitive photopigment, is the best studied, mainly because it is the only neuropsin found in mammals. In *Xenopus, opn5* is present in cells of the inner nuclear and ganglion cell layers (Figure 3A; Yamashita et al., 2014; Bertolesi et al., 2021), as well as deep in the brain (Currie et al., 2016). Assuming some evolutionary conservation in Opn5 function, its presence in the *Xenopus* retina likely involves non-visual roles. In support, mammalian OPN5 regulates the photoentrainment of the retina (Buhr et al., 2015), with *Opn5* null mice showing impaired photoentrainment and phase shifting to UVA light (Ota et al., 2018). Of note, the deep brain diencephalic neurons that express *opn5* may participate in motor/behavioral responses. For instance, a role for Opn5 in light-dependent swimming, independent of the eye and the pineal complex, was suggested in *Xenopus*, although genetic support is lacking (Currie et al., 2016). In birds, hypothalamic *opn5* positive cells are thought to contribute to migration and seasonal reproduction (Nakane et al., 2014). The role of other retinal-expressed neuropsins, such as Opn6 and Opn8, is unknown. *opn8* is expressed by INL cells of the *Xenopus* retina, likely amacrine cells, while *opn5* mRNA may be present in bipolar cells (Bertolesi et al., 2021; Figure 3A). Finally, *opn6* is expressed by newly born photoreceptors in the ONL of the peripheral retina bordering the proliferative ciliary marginal zone (CMZ) (Figure 3A; Bertolesi et al., 2021).

Most of the opsins, including all of the photoreceptor opsins, bind 11-*cis* retinal in their resting state. 11-*cis* retinal is isomerized to all-trans retinal following light activation. Thus, a functional retina requires a continuous supply of new 11-*cis* retinal. The RPE functions as the supplier for the reconversion of all-*trans* to 11-*cis* retinal, which is mediated by two photoisomerases, RGR and RRH (Bellingham et al., 2003; Davies et al., 2015). In *Xenopus* larvae, both photoisomerases are expressed by the RPE outside of the CMZ (Bertolesi et al., 2021; Figure 3A) as befits their supplier role to photoreceptors. Yet, it is unclear if RGR and RRH are expressed by the same or different RPE cells. An interesting yet controversial issue is the source of 11-*cis* retinal for opsins present in cells located at a distance from the RPE. A possible explanation is provided by the biochemical characteristics of Opn5 of *Xenopus* and birds. In these species, the Opn5 protein binds 11-*cis*-retinal to produce a UV light-absorbing form, but also binds all-trans-retinal to produce a visible light-absorbing form that generates 11-*cis* retinal. Cycling between UV- and yellow light reconverts one form to the other repeatedly (Yamashita et al., 2014; Sato et al., 2018b). Further studies are necessary to determine if Opn5, 6, 7, and 8 may work as bidirectional forms of opsins, or function to provide 11-*cis* retinal to other opsins located away from the RPE.

The maximum absorption peaks of the *Xenopus* opsins as compared with human visual opsins is shown in Figure 3B. The rods include those that express either green-sensitive Rhodopsin (λ max ∼ 500–523 nm) (Dartnall, 1956; Batni et al., 1996) or blue-sensitive Sws2 (λ max ∼ 434 nm) (Darden et al., 2003). The most abundant cone, expressing Lws, is red sensitive (λ max ∼ 611 nm) (Witkovsky et al., 1981) and shows peak sensitivity at a longer wavelength than its human counterpart, while the Sws1 cone is blue-violet sensitive (λ max ∼ 425 nm) (Starace and Knox, 1998; Figure 3B). Pinopsin and melanopsins (Opn4 and Opn4b) have maximum absorbances at 470 nm and approximately 480 nm, respectively (Shirzad-Wasei and DeGrip, 2016; Sato et al., 2018a). It is important to note that the absorbance spectrum of opsins changes depending on the chromophore used (Vitamin A1 or A2). For example, *Xenopus* Rh1 shifts from 500 to 523 nm and Sws2 from 434 to 443 nm with the use of Vitamin A1 or A2, respectively (Dartnall, 1956; Witkovsky et al., 1981; Darden et al., 2003). The mammalian chromophore, Vitamin A1, is the form used primarily in experiments analyzing the physicochemical properties of type II opsins. In amphibians, however, the main chromophore is Vitamin A2, which shifts the spectrum to a longer absorbance. Indeed, adult *Xenopu*s retina contains only 5–12% Vitamin A1 (Wald, 1955) and Vitamin A2 is almost exclusively detected in stage 43/44 retinas (Bridges et al., 1977).

The onset of retinal function, which requires the developmental expression of type II opsins, contributes to our understanding of the mechanism that regulates changes in skin pigmentation. Cones are both born and determined before the first rods appear (Chang and Harris, 1998). Interestingly, while both rods and red cones begins to emerge early in development (stage 23; 1.5 day at 16°C), the expression of the classical type II opsins initiates in most photoreceptors only a day later at stage 32, and becomes pronounced by stage 35/36 (Figure 3A). Melanopsin expression in both ipHCs and ipRGCs, like opsins in classical photoreceptors, initiates by stage 32 and is readily detectable at stage 33/34 (Bertolesi et al., 2014; Figure 3A). Our understanding of when opsin expression initiates is based on the more abundant rods and cones: Rh1- and Lws- expressing, respectively (Stiemke et al., 1994; Chang and Harris, 1998). Likely the timetable for generating photoreceptors expressing other opsins is similar, with the exception of pinopsin-expressing cones that emerge at stage 42/43 (Bertolesi et al., 2021). Photoreceptors first form synapses at stage 37/38 (Witkovsky, 2000), and this is when the retina becomes light sensitive. Using light-induced *c-fos* expression, we defined functional ‘unit circuits’ at stage 37/38 that consist of two to three *c-fos* positive INL cells and one RGC, which increase over time (Bertolesi et al., 2014). How do these results correspond with the emergence of the eye-dependent background adaptation response? The initial studies of background adaptation performed by Hogben and Slome (1936) suggest a differential activation of the retina, with the dorsal part capturing light reflected from the surface. Interestingly, pinopsin and melanopsin are both located in the dorsal retina (Figure 3A). The onset of pinopsin expression in the eye, however, occurs a day after background adaptation is detected (stage 39/40) (day 4 in Figures 1A, 3A; Bertolesi et al., 2021). Thus, pinopsin is eliminated from a list of potential light sensors for background adaptation. Instead, melanopsin (ipHCs or ipRGC) may be involved; Indeed, melanopsin mRNA appears prior to the phenotypic response (Bertolesi et al., 2014), and pharmacological inhibition of melanopsin (AA92593) in the eye induces the synthesis and release of α-MSH and consequent skin darkening (Bertolesi et al., 2015, 2016b). Together, the data support a model where the dorsally located melanopsin-expressing cells are the photosensors for background adaptation. The circuit that links light sensitivity in the eye to the release of α-MSH from the pars intermedia pituitary involves an intermediary, the suprachiasmatic melanotrope inhibitory neuron (SMIN) (Ubink et al., 1998). The model proposes that with a white background the dorsal retina receives more light than the ventral retina, because of the reflected light from the surface. Activation of the ipHCs and/or ipRGCs, and subsequently the intermediary SMIN neurons, blocks the synthesis and release of α-MSH from melanotropes located in the hypophysis. Conversely, with a black background the ipHCs/ipRGCs are inactive, releasing the inhibition on α-MSH secretion and inducing melanosome dispersion and skin darkening (Bertolesi and McFarlane, 2018). An open question is if ipHCs and/or ipRGCs have similar or different roles in the background adaptation process. Both cell types are preferentially located in the dorsal retina. The ipHCs are seven time more abundant than ipRGCs (Bertolesi et al., 2014), however, and in birds ipHCs act as photoreceptors (Morera et al., 2016). Nonetheless, more studies are necessary to identify the melanopsin expressing cell that serves as photoreceptor during background adaptation.

The delayed developmental expression of pinopsin relative to visual opsins suggests a role in a behavioral response associated with background recognition. Indeed, older tadpoles (stage 45/46) are able to discriminate different colors and intensities of light, including those generated by different substrates (Rothman et al., 2016; Hunt et al., 2020). Interestingly, young tadpoles adapted to a white background prefer this surface, while froglets choose a black background irrespective of their previous adaptation (Moriya et al., 1996a; Bertolesi et al., 2021). Changes in pinopsin retinal expression during development correlates with this behavioral response (Bertolesi et al., 2021). Together, these data suggest that opsins like melanopsin (Opn4 and Opn4b) and pinopsin that are linked classically to non-visual roles in mammals (sleep and circadian rhythm) and birds (melatonin secretion) also function in background recognition in young tadpoles *via* the eye.

### Structure of the Pineal Complex, Expression of Type II Opsins and Melatonin Secretion

In *Xenopus*, type II opsins expressed in the pineal complex sense light that regulates melatonin secretion, and therefore, a non-visual response in skin pigmentation. The first evidence for this pathway was that ‘pineal gland’ extracts caused lightening in amphibian tadpoles (McCord and Allen, 1917). This pigmentation response was then used as a biological readout during melatonin purification from the mammalian pineal gland (Lerner et al., 1958). Structurally, the pineal gland is integrated in the brains of mammals. In amphibians, the gland is referred to as the pineal complex as it consists of two components: (1) the pineal gland, which is the neuroendocrine-secretory organ, and (2) the frontal organ, an accessory photosensitive structure (Charlton, 1968; Korf et al., 1981; Sapède and Cau, 2013). Of note, the frontal organ is present in many non-mammalian vertebrates, although it is given different names, such as the parapineal organ in teleosts and lamprey, and parietal eye in non-avian reptiles (Sapède and Cau, 2013). The pineal complex displays a simpler structure than the retina, containing only two neuronal types, photoreceptors and projection neurons. These two cells are generated from the same pool of floating head precursors and represent the functional homologs of retinal photoreceptors and retinal ganglion cells, respectively (Arendt, 2003; Sapède and Cau, 2013). In adult frogs, the pineal gland is located intracranially, at the top of the brain, while the frontal organ sits extracranially in close proximity (Charlton, 1968; Korf et al., 1981; Sapède and Cau, 2013). Anatomically, the pineal complex can be visualized in stage 42 *Xenopus* larvae, with the photosensitive frontal organ located under the skin and the pineal gland sitting immediately beside it in a dorsal position between the habenular and posterior commissures (Bertolesi et al., 2020). *in situ* hybridization and immunohistochemistry analyses reveal that the pineal complex is functional in *Xenopus* larvae by stage 43, with both the frontal and pineal organ displaying phenotypic characteristics of sensory and secretory tissues (Bertolesi et al., 2020). It is worth noting that at stage 43/44 the two structures are not completely separated. Additionally, the intra- and extra-cranial tissues are not yet evident, since the formation of bony-skull structure initiates at stage 52 (Slater et al., 2009).

The pineal complex primarily regulates sleep and wake cycles by transducing light and dark information and subsequently melatonin secretion. Light establishes the pattern by which melatonin is secreted by the pineal gland, with melatonin at low levels during the day and peaking at night (Coomans et al., 2015). The release of melatonin also influences both thermoregulation and the control of daily and seasonal rhythmic processes (Filadelfi and Castrucci, 1996; Simonneaux and Ribelayga, 2003; Nisembaum et al., 2015). Interestingly, recent studies show that type II opsins detect not only light, but thermal energy can act *via* the opsins to induce cellular activity (reviewed by Moraes et al., 2021). The thermal contribution of type II opsins to melatonin secretion, and thus skin pigmentation, has not yet been investigated and is not addressed in this review.

In vertebrates, melatonin synthesis occurs in four enzymatic steps, with the third one critical for light-regulated rhythmicity (Falcón et al., 2009). The third step is the conversion of serotonin to *N*-acetylserotonin by aryl alkylamine *N*-acetyltransferase (AANAT), and then a final *O*-methylation of *N*-acetylserotonin by hydroxyindole-*O*-methyltransferase to produce melatonin (Falcón et al., 2009). Interestingly, AANAT plays a significant role both in the synthesis of melatonin and in driving the rhythmic activity of melatonin release from both the pineal gland and the eye (Klein, 2006). Indeed, this key enzyme itself is regulated at the transcriptional and post-translational levels in a circadian manner, as levels are low during the day with activity peaking at night (Klein, 2006). In frogs, Aanat is expressed in retinal photoreceptors as well as in the pineal epithelium where pineal photoreceptors reside. Aanat labeling is concentrated in the inner segment in contact with the cerebrospinal fluid in the pineal lumen (Isorna et al., 2006), suggesting that the pineal photoreceptors possess both secretory and photoreceptor functions. In concordance with Aanat being present in both the *Xenopus* eye and pineal complex, melatonin is synthesized in both structures (Wiechmann and Sherry, 2012). Retinal melatonin may exert physiologically insignificant effects on pigmentation, however, given its rapid degradation (Green et al., 1999). Indeed, retinal melatonin behaves as a local neuromodulator, while pinealectomy results in a significant decrease in or complete abolition of plasma melatonin (Cahill and Besharse, 1989). In agreement, pinealectomy, but not enucleation, affects pigment aggregation induced by dark (Figure 1B).

Developmentally, *Xenopus* embryos express the enzymes necessary for melatonin biosynthesis, and ‘pineal’ *in vitro* explants show melatonin production as early as stage 26 (approximately 30 h post-fertilization), despite the pineal complex itself evaginating from the diencephalon on the second day of embryogenesis (Green et al., 1999). Interestingly, differentiation of neurons in the habenular nuclei, a paired structure anterior to the pineal organ, is upregulated by light exposure and reduced by melatonin treatment in early development (36 h post fertilization) of zebrafish (de Borsetti et al., 2011). At an equivalent developmental time in *Xenopus*, Bsx, a transcription factor that controls pineal progenitor proliferation and the photoreceptor fate, is also expressed with light rhythmicity (D’Autilia et al., 2010). This raises the question as to what photosensor/s senses the light that induces both the expression of Bsx and the melatonin-synthesizing Aanat enzyme? Additionally, when does expression of the photosensor initiate? In contrast to what is known of roles for type II opsins in the *Xenopus* retina, our understanding of pineal opsins is limited. mRNAs of type II opsins such as *opnp, opnpp*, *opnpt*, *opn3*, *opn4*, *opn4b*, *opn5*, *opn6b*, and *rgr* (Figure 2) are detected in the *Xenopus* pineal complex at stage 43/44 (Bertolesi et al., 2020). Data from our laboratory suggests that the pineal complex becomes fully functional to induce skin lightening at stage 39 (Figure 1A). Cells of the pineal complex in stage 43/44 *Xenopus* larvae exhibit sensitivity to environmental light conditions, with lightening of the skin occurring 30 min after a piece of tin foil is placed atop the pineal to prevent light from reaching the complex (Bertolesi et al., 2020). It is possible that while melatonin-synthesizing cells can produce melatonin at early developmental times, as suggested by the *in vitro* studies in *Xenopus* (Green et al., 1999), and zebrafish (de Borsetti et al., 2011), it is not until later that the cells produce sufficient melatonin to affect skin pigmentation. The opsin sensor expressed at early stages is currently unknown and further studies are necessary to determine the onset of pineal opsin expression.

A comparative analysis of opsins expressed by the *Xenopus* pineal complex with other species, as well as their wavelength sensitivity, has proven helpful for understanding the evolutionary functionalization of opsins and their possible role in regulating melatonin secretion, and therefore skin pigmentation. The nerves of the frontal organ in adult *X. laevis* show light sensitivity with both achromatic and chromatic responses. Two separate mechanisms underlie the chromatic response: The inhibitory response is maximally sensitive at approximately 360 nm (UV) while the excitatory response peaks at 520 nm. In comparison, the action spectrum of the achromatic response of the pineal and frontal organs peaks between 500 and 520 nm (Korf et al., 1981). A plethora of opsins are expressed in the stage 43/44 pineal complex (Figure 2). Of these, parapinopsin (Opnpp) and Opn5 likely function as absorbers of UV light in *Xenopus* and other vertebrates (Wada et al., 2012; Yamashita et al., 2014), and therefore could drive the inhibitory response at 360 nm. However, none of the UV-sensitive opsins are likely involved in the lightening of skin pigmentation, in that such a response in *Xenopus* is inhibited by visible-light (maximum inhibition between 470 and 650 nm) (Bertolesi et al., 2020). In teleosts, melatonin release from a perfused pineal is inhibited maximally at 520 nm but is not impacted by UV light (Ziv et al., 2007). Only one parapinopsin gene exists in *Xenopus* with the second lost likely by pseudogenization. In the teleost lineage, however, the parapinospin gene was duplicated during the teleost specific genome duplication. The spectral sensitivity and expression pattern of the paralogs differ, with one remaining as UV-sensitive (PP1) while the other became a blue-light sensitive opsin (PP2) (Koyanagi et al., 2015), reflecting the functionalization of parapinopsins during teleost evolution. Additionally, parietopsin (Opnpt) has a maximum absorbance (522 nm) corresponding to the peak of the achromatic excitatory signal in frogs (Su et al., 2006). Interestingly, the parapinopsin and the parietopsin genes disappeared in avian reptiles and turtles concomitant with the loss of the parietal eye in these lineages (Emerling, 2017), reinforcing the idea of a role for these opsins exclusively as frontal organ sensors. As in *Xenopus*, contraposed achromatic responses are described for the pineal complex of lamprey and teleosts, with an inhibitory response mediated by UV-light and an excitatory one by visible light (520 to 560 nm), and assigned to parapinopsin and parietopsin sensitivity, respectively (Koyanagi et al., 2015, 2017; Wada et al., 2021). Within the pineal complex, the encephalopsin (Opn3) as well as the melanopsins (Opn4 and Opn4b) function as blue light-sensitive opsins, while Opn4 acts as a light sensor in the mammalian eye that regulates melatonin secretion and circadian rhythm entrainment (Prayag et al., 2019). The role of the neuropsin Opn6b remains poorly understood, while the retinal G-protein coupled receptor Rgr functions as a photoisomerase in the pineal complex, binding trans-retinal for chromophore conversion. Taken together, several opsins sensitive in the visual range of the light spectrum may serve as the photosensors that mediate skin lightening by the pineal complex. However, a complete understanding of their roles in skin pigmentation still remains to be determined.

### Type II Opsins in the Skin. From the Ectothermic *Xenopus* to Endothermic Mammals

In addition to the eye and the pineal complex, the skin/integument of animals is an essential light sensor as it is exposed directly to environmental light. While the role that photosensation plays in different skin cells is not well understood, in general, UV protection and circadian regulation are considered the two most important functions (Slominski et al., 2004; Sapède and Cau, 2013; Bertolesi and McFarlane, 2018). A better understanding of how type II opsins regulate melanosome movements in *Xenopus laevis* melanophores will contribute to our knowledge of how the only pigment producing cells of human skin, the melanocytes, respond directly to sunlight, in that melanocytes derive evolutionarily from melanophores of the amphibian ancestors (Ligon and Mccartney, 2016; Schartl et al., 2016; McNamara et al., 2021). Evolutionary, human skin has conserved a cell-autonomous mechanism to regulate the expression of circadian clock genes (Hardman et al., 2015). Melanocytes secrete melanin-containing vesicles to neighboring cells, keratinocytes, which retain the melanin. The secretory phenotype of the melanocytes likely emerged when fur and feathers evolved as insulation systems for thermoregulation. Two important observations support this idea: (1) The number and diversity of melanosomes detected in well-preserved fossils of avian reptiles and mammalian lineages increase in correlation with the emergence of thermoregulation (Lindgren et al., 2015; Lovegrove, 2017). These phenotypic differences remain in existent melanophores and melanocytes of amphibians and mammals, respectively; and (2) Pheomelanin, a derivative of melanin, is synthesized in melanocytes of birds and mammals, but not fish (Kottler et al., 2015; McNamara et al., 2021). Interestingly, pheomelanin is also detected in amphibian fossils (Colleary et al., 2015). Of note, similar to other ectotherms, *Xenopus laevis* has additional types of chromatophores, including iridophores that reflect light and give the skin a shiny appearance, and xanthophores, which appear yellow or orange due to the presence of pteridines (Nakajima et al., 2021). However, these cell types appear later in development than melanophores (Droin, 1992).

Opsin proteins expressed by skin cell melanophores relay environmental light information to the cell. With the physiological regulation of skin color change in *Xenopus* tadpoles (discussed above), the primary response mediated by direct action of light is not as important as the secondary response mediated by the neuroendocrine system. However, direct responses to light and dark through melanosome aggregation and dispersion are present in some amphibian species, including *X. laevis* (Moriya et al., 1996b; Oshima, 2001). Interestingly, while three different melanophore cell lines established from *Xenopus laevis* show a common dispersing and aggregating response to α-MSH and melatonin (secondary response), respectively, they exhibit distinct responses to direct light. Two cell lines were established by isolating melanophores from stage 30 to 35 *Xenopus* tadpoles. These cell lines, named MEL (Daniolos et al., 1990) and MEX (Kashina et al., 2004), aggregate melanosomes in the dark and disperse them with light. In MEL cells, light induces melanosome dispersion at 460 nm in the presence of all-trans retinal (Daniolos et al., 1990; Rollag, 1996), suggesting an opsin-mediated response. At least two opsins, Opn4 and Opn4b, are present in MEL cells (Moraes et al., 2015), while several opsin genes are expressed in MEX cells, including the melanopsins (Figure 2). Interestingly, Opn4 and Opn4b show a distinct subcellular localization, with Opn4b found in the cell membrane and cytoplasm, and Opn4 in the nucleus (Moraes et al., 2015). While we know by RT-PCR analysis which opsin genes are expressed in the skin (Figure 2), we do not know in which cell types the genes are transcribed and whether protein is present. The MEX cells allow us to directly assess expression of opsins by melanophores: RT-PCR analysis indicates mRNA for *sws2, tmtops, tmtopsb, opn5*, *opn7a/b, opn8*, *opn4, and opn4b*. Given the diversity of opsin expression in melanophores, assigning a function to melanopsin or any other opsin as a physiological sensor during the light-induced dispersion of melanosomes in MEL and MEX is not yet possible. The third melanophore cell line was generated from isolated tail fins of *Xenopus* tadpoles between stages 51–54 (Seldenrijk et al., 1979). These cells also show melanosome dispersion in response to α-MSH. In contrast to MEL and MEX cell lines, however, these tail fin melanophores aggregate their melanosomes in light and disperse then in the dark, with approximately half of the melanophores in the dish exhibiting light sensitivity (Seldenrijk et al., 1979). This light-mediated aggregation response concurs with that observed in isolated tail fins from *Xenopus* larvae at the same developmental stage (Moriya et al., 1996b). Similarly, melanosome aggregation mediated by light is observed during tail regeneration in stage 49 and older tadpoles (Bagnara, 1960b). The differences in the primary response to light of these cultured melanophores obtained at different developmental stages may be due to the differential expression of opsins in melanophores. Of note, our RT-PCR analysis of opsins from MEX cells (stage 30–35) and isolated tails from stage 43/44 indicate differences (Figure 2), suggesting that dynamic opsin expression occurs over development. Why there are changes in the primary light response as the tadpoles age, and which opsins are expressed by the melanophores of pre-metamorphic tadpoles, still needs answering.

Initial studies performed in isolated fin tails or cultured melanophores from *Xenopus laevis* allowed authors to suggest a possible role of the visual opsins, particularly rhodopsin, in pigment cells (Daniolos et al., 1990; Miyashita et al., 1996; Moriya et al., 1996a). These studies identified rhodopsin by immunolabeling and by spectral analysis of the melanophore response to light. The extent of the diversity of opsins, however, was not known at that time, and brings into question the specificity of the antibodies that were used to recognize the visual rhodopsin. Indeed, our analysis of opsin gene expression by RT-PCR in tails isolated from stage 43/44 *Xenopus* shows no expression of the classical visual opsins (Figures 2A,B). In agreement, in MEX cells only *sws2* is consistently detectable (Figures 2A,B). Melanopsin *opn4* and *opn4b*, and the *opn5* neuropsin are thought to be critical for circadian regulation and UV protection in mammalian melanocytes. For example, OPN5 induces clock gene expression and circadian photoentrainment in mouse melanocytes (Buhr et al., 2019), and participates in UV-mediated melanogenesis in human melanocytes *in vitro* (Lan et al., 2021). OPN4 also participates in melanogenesis of mammalian melanocytes, as well as controlling melanocyte proliferation (de Assis et al., 2018, 2020). The presence of Opn4 protein (Provencio et al., 1998) and *opn5* mRNA in tail skin and melanophores (Figures 2A,B) indicates that these proteins may play comparable roles in *Xenopus*. *Opn7a/b* and *opn8* do not exist in avian reptiles and mammals, but are expressed in the *Xenopus* tadpole tail and MEX cells (Figure 2), though with unknown function. There are no reports of pineal opsins regulating mammalian melanocytes. Thus, it was not surprising that, with the exception of low expression of VA-opsin in the tail, we did not detect pineal opsin expression in the tail or MEX cells (Figure 2). *Opn3* is absent from *Xenopus* tails and MEX cells at early developmental times (Figure 2), but expression may occur later during development, as OPN3 regulates pigmentation of mammalian melanocytes (Ozdeslik et al., 2019; Olinski et al., 2020) Alternatively, through evolution the expression of *opn3* may have been lost in *Xenopus* melanophores. Finally, a family of opsins, whose ubiquitous expression in zebrafish gave them the name of *teleost multiple tissue opsins* (tmtops), regulate the entrainment of the peripheral circadian clock (Moutsaki et al., 2003). Of the three *tmt* opsins found in the *Xenopus* genome (*tmtops*, *tmtops2*, and *tmtopsb*), two of them, *tmtops* and *tmtopnb*, are expressed in the tail and in MEX cells (Figure 2). Interestingly, genes of the *tmt* family remain in almost all vertebrate genomes, including avian reptiles and mammals (monotremes and marsupials). *tmt* genes did disappear recently in the eutherians (Davies et al., 2015), suggesting that the evolutionary selection of *tmt genes* was unaffected by thermoregulation and the melanophore/melanocyte transition. Together, these data show that many but not all of the opsins present in *Xenopus* melanophores remain in mammalian melanocytes with an assigned role either in the cell autonomous peripheral entrainment of the skin cell cycle or as a UV/light regulator of melanogenesis.

## Conclusion

In *Xenopus laevis* tadpoles, light sensed by the eye, the pineal complex and the skin produces specific physiological responses that include changes in skin pigmentation. For example, the color of the background surface is sensed by the eye to produce changes in skin pigmentation to prevent the tadpoles from being detected by predators. This cryptic response, named background adaptation, increases survival. In contrast, detection of environmental day light by the pineal complex is used to alter skin pigmentation levels to adjust for thermoregulation or UV protection. The photosensors for these physiological responses are type II opsins, with their roles just beginning to be elucidated. A large number of diverse type II opsins exist in vertebrates, which are expressed in different organs. In *X. laevis* we identified 22 separate opsin genes, after removal of the duplicated forms characteristic of the allotetraploid nature of this species. In the last few years, we analyzed the expression of these type II opsins. These expression data we combined with skin pigmentation assays to establish for distinct pigmentation responses the participating light-sensitive organ and type II opsin(s) that works as the light sensor.

In the Anuran eye, the classical visual opsins are expressed by two rods (*rh1* and *sws2*) and two cones (*lws* and *sws1)*. Additionally, *pinopsin* is co-expressed with *sws1* in a few dorsally located cone photoreceptors. The melanopsin genes (*opn4* and *opn4b*) are also expressed dorsally in ipHCs. These ipHCs likely act as “photoreceptors” to regulate background adaptation, with pharmacological inhibition of melanopsin increasing α-MSH release from the pars intermedia pituitary to darken the skin. In contrast, we propose that pinopsin transduces the surface color to initiate a behavioral response where the organism chooses a specific background surface (background preference). A role for ipHCs as “photoreceptors,” and the expression of more than one opsin in eye classical photoreceptors (e.a. *pino* + *sws1*+) (Morera et al., 2016; Sato et al., 2018a; Bertolesi et al., 2021), are novel discoveries that likely will push *Xenopus laevis* forward on a new retinal research journey. Additional opsins, such as the neuropsins, *opn5*, *6*, and *8*, are expressed in the eye, although their function is not known.

*Xenopus* tadpoles lighten the skin in the dark because at night the pineal complex increases its secretion of melatonin into the circulation. Skin lightening and melatonin release are inhibited maximally by 520 nm visible light, which alongside the expression data, points to the involvement of candidate opsins. Several opsins expressed in the pineal complex absorb light at this wavelength, including Opn4, Opn4b, Opn3, Pinopsin (*opnp*) and Parietopsin (*opnpt*). Interestingly, *parapinopsin* (*opnpp*) and *parietopsin* are expressed in the frontal organ, and their UV and blue/green light sensitivities, respectively, correlate with the inhibitory and stimulatory electrophysiological recordings detected in the nerve of the adult frontal organs (Korf et al., 1981). During evolution, *parapinopsin* and *parietopsin* genes disappeared in avian reptiles and turtles concomitant with the loss of the parietal eye in these lineages (Emerling, 2017). The use of CRISPR/Cas9 technology to knock out different opsins in F0 generation *Xenopus* (Blitz and Nakayama, 2021), in combination with pigmentation assays, will help provide an answer to the question as to whether one or more pineal complex-expressed opsins regulate melatonin secretion.

Finally, we analyzed the mRNA expression of *Xenopus* opsins in the tail and in a melanophore cell line (MEX cells) and compared the expression with what we currently know of their role in mammalian melanocytes. Several opsins, such as Opn4 and Opn5, have been evolutionarily selected as a sensor for either the cell autonomous entrainment of the melanocyte cell cycle or for UV/light regulation of melanogenesis. These genes are conserved between melanophores and melanocytes.

Studying the regulation of skin pigmentation in *Xenopus laevis* has proven extremely insightful for our understanding of photosensory organs (eye, pineal complex, and skin) and their evolution. The recent full sequencing of the *Xenopus laevis* genome, the discovery of several type II opsin photopigments, and novel technologies have established this model organism for new fields of investigation.

## Author Contributions

GB and ND performed the RT-PCR study from tails and MEX cells. GB, ND, HM, LM, and SM wrote the manuscript and critically revised the manuscript. All authors contributed to the article and approved the submitted version.

## Conflict of Interest

The authors declare that the research was conducted in the absence of any commercial or financial relationships that could be construed as a potential conflict of interest.

## Publisher’s Note

All claims expressed in this article are solely those of the authors and do not necessarily represent those of their affiliated organizations, or those of the publisher, the editors and the reviewers. Any product that may be evaluated in this article, or claim that may be made by its manufacturer, is not guaranteed or endorsed by the publisher.

## Footnotes

1
http://www.xenbase.org/entry/
